# Use of Food Services by Consumers in the SARS-CoV-2 Pandemic. How the Eating Habits of Consumers Changed in View of the New Disease Risk Factors?

**DOI:** 10.3390/nu13082760

**Published:** 2021-08-11

**Authors:** Ewa Czarniecka-Skubina, Marlena Pielak, Piotr Sałek, Artur Głuchowski, Joanna Kobus-Cisowska, Tomasz Owczarek

**Affiliations:** 1 Department of Food Gastronomy and Food Hygiene, Institute of Human Nutrition Sciences, Warsaw University of Life Sciences (WULS), Str. Nowoursynowska 166, 02-787 Warsaw, Poland; marlena_pielak@sggw.edu.pl (M.P.); piotr_salek@sggw.edu.pl (P.S.); artur_gluchowski@sggw.edu.pl (A.G.); 2 Department of Gastronomy Sciences and Functional Foods, Faculty of Food Science and Nutrition, Poznan University of Life Sciences, Wojska Polskiego 28, 60-637 Poznań, Poland; joanna.kobus-cisowska@up.poznan.pl; 3 Department of Management and Economics, Gdynia Maritime University, Str. Morska 81-87, 81-225 Gdynia, Poland; t.owczarek@wzjn.umg.edu.pl

**Keywords:** food service, consumer, eating habits, SARS-CoV-2, Poles

## Abstract

The SARS-CoV-2 pandemic in 2020–2021 changed the eating habits of people around the world. The aim of this study is to understand the effects of COVID-19 on changing consumers’ eating habits, including their concerns about food service nutrition in case of new disease risk factors. The survey conducted using the computer-assisted web-based interviewing method on a group of 1021 adult respondents in Poland. We collected information about consumer choices and habits related to use of food services during the pandemic. This research found that COVID-19 had an impact on consumers’ use of food services, both on-site and take-away. Using cluster analysis, we identified five main groups of food service consumers. It was found that almost half of the respondent group did not change their diet during the pandemic, 20% of respondents changed their diet to a positive one, and 20% to a diet that was negative. For respondents the most important forms of protection against COVID-19 in catering establishments were hand disinfection (70.3%), table disinfection (70.4%), wearing of masks and visors by staff (68.2%), and the possibility of cashless payments (64.6%). Based on cluster analysis (eight consumer clusters), we stated that majority of respondents did not see any threats to using catering service during the pandemic. Only a small group (8.1%) of respondents were afraid of the possibility of getting sick with COVID-19. This study presented the effects of COVID-19 on consumer eating behavior in catering and their concerns with food services uses. Discovering consumer concerns can reduce risk, increase food safety and improve eating habits.

## 1. Introduction

COVID-19 disease, caused by SARS-CoV-2, was first reported in December 2019 in Wuhan, China. Symptoms of the disease develop from 2 to 14 days after exposure, causing fever, cough, and shortness of breath, sore throat and muscles, and loss of taste and smell [1,2]. It may also be asymptomatic, have mild or severe symptoms leading to hospitalization and even death in patients with comorbidities [3,4,5,6]. Person-to-person transmission of the virus occurs at local, regional, national, and international levels. The virus can make others sick and contaminate the environment, including food products [7]. The virus spreads rapidly around the world, which is why in March 2020 a global pandemic was announced. Countries (e.g., China, South Korea, India) where the first cases of the disease were confirmed began to implement a rigorous hygiene regime and nationwide prevention measures such as disinfection, personal protective equipment, complete restriction on all international and domestic travel, social isolation, and suspension of many services and quarantine. Companies that were essential to society or offering basic necessities, such as grocery stores, remained open to consumers while companies and institutions that gather a lot of people, such as restaurants, hotels, and various types of schools, were temporarily closed [8,9], except take-away and home deliveries. About 4 billion people have been forced by the pandemic to either quarantine or isolate themselves at home [10,11,12]. The COVID-19 pandemic has severely impacted the global economy with huge effects on all business sectors, including tourism, food service, cultural events, and trade fairs [13].

In March 2020, the gastronomy and hotel industry, shopping malls, schools, and universities were all closed in Poland, introducing a remote education system and restrictions applied to public meetings. For most Poles, the initial order to stay at home was for six weeks. For selected groups, this period was longer and covered more than one year [14,15]. This has led to a noticeable decrease in “social consumption”. The gastronomy and hotel industry suffered the most because of the introduced restrictions. The average decrease in demand for goods and services in the catering and accommodation industry during pandemic (April 2020–February 2021) dropped −41.4% [16,17]. The Polish Gastronomy Chamber of Commerce estimates that in April–May 2021, 15,000 business have been bankrupted due to the lockdown of catering establishments (i.e., about 20% of all facilities). The pandemic stopped the previous clear trend of increasing household expenditure on food services in Poland [18]. The effects of the current SARS-CoV-2 will be the most clear and durable in the tourism and hospitality sectors, as habits related to traveling and eating changed [19]. The pandemic resulted in huge revenue losses in the catering industry, accounting for up to 80% of revenue, and many restaurants were closed, with even fast-food sales in the U.S. and China decreasing [20,21,22,23]. Similar changes were found in Europe [24].

Predicting trends, analyzing customer needs and consumer behavior, by studying the factors that influence consumer choice and restaurant demand, is particularly important for the catering industry, due to the instability of related products and services, and may be helpful in restaurant management [25]. However, the COVID-19 pandemic differs from other food organization crises because of its severity, devastating effects, evolving nature, and very limited organizations’ ability to control the situation. Due to the nature of the COVID-19 crisis, the need for strategic action and searching for new channels for the provision of food and catering services is a key aspect for catering organizations.

Discovering consumer concerns can reduce risk, increase safety, and improve eating habits. The aim of this study is to understand the effects of COVID-19 on changing consumers’ eating habits, including their concerns about food service nutrition in case of new disease risk factors. The second goal of this study is to identify, describe, and compare consumer segments based on differences in individual food choices, concerns, and eating habits in catering establishments during the COVID-19 pandemic.

## 2. Literature Review

Human disease pandemics are influencing consumer psychology and consumer behavior and habits. The international crisis caused by the spread of the new coronavirus has brought food safety concerns to the fore. Risk perception plays a key role in determining healthy behavior [26]. Anxiety caused by the food shortages in grocery stores triggered panic in customer, who focused on buying semi-finished products and food products to prepare at home [27]. Restrictions on restaurants were one of the reasons for the excess accumulation of products by consumers.

Quarantines and restrictions related to the COVID-19 pandemic can be considered highly stressful events that affect eating patterns. The transition period associated with a pandemic situation, therefore, has the potential to change eating habits, forcing most people to stay at home, sometimes in home-offices, for long periods, often with unlimited access to food in home and less physical activity [28]. Staying at home for a long time may favor snacking between meals, eating ‘junk food’ snacks, and drinking alcohol, and it may affect the consumer’s individual choices about cooking at home, buying ready-to-eat products, as well as using takeaway food services [28,29,30,31,32,33,34,35,36]. The perceived risk of COVID-19 and guidelines for minimizing personal contact might have been discouraging consumers from using gastronomy during the pandemic [37,38].

In the literature on the functioning of gastronomy and consumer behavior before the SARS-CoV-2 pandemic, numerous studies examine factors influencing the demand for catering establishments, such as: food quality [39], hygiene and food safety [40], price [41], quality of services [42], location [43], and online consumer feedback [44]. Crise such as the pandemic 2020/2021, constitute a unique, critical force among the mentioned factors and affect consumer behavior and the functioning of the foodservice industry, creating serious consequences for the industry. Before the COVID-19 pandemic, only a few empirical studies [45,46,47,48,49,50,51] looked at the impact of crises on consumer food choices, demand in restaurants and managing food production during crise caused by epidemic diseases, such as severe acute respiratory syndrome (SARS) and the avian flu. Few studies [52] concerned the reactions of consumers to the crises. Most of the research looked at the impact of food safety and economic crises on the foodservice industry. However, there was a lack of empirical studies that took into account the impact of the epidemic crisis on the demand and operation of restaurants [53] and on consumer behavior related to nutrition in gastronomy. Some studies indicate the socio-economic effects of pandemic on individual aspects of the world economy [54,55,56,57,58]. Many previous research papers [14,28,32,33,34,35,53,59,60,61,62,63,64,65,66,67,68,69,70,71] conducted during the pandemic were devoted to the impact of the situation on eating habits, adherence to a the Mediterranean diet in Italy, a potential protective role of micronutrients, phytochemicals and Mediterranean diet against COVID-19, the potential to increase the stress level and its associated impact on the pre-existing diseases such as diabetes and changes in the diet of people or the impact of nutritional status in patients with COVID-19 and those who have had the disease [61,72,73,74]. So far, only a few studies [31,63] have analyzed the impact of the pandemic on use of food services.

We are supplementing the literature by examining the effects of COVID-19 on consumer eating habits in catering and their possible concerns in the light of new disease risk factors.

## 3. Materials and Methods

### 3.1. Study Design

This paper was designed as a study with a convenience sampling. The respondents completed the questionnaire online. A link to the questionnaire in a Polish language Google Forms format was sent via Facebook, WhatsApp, e-mail, and students’ forum. A questionnaire provided on a webpage increases the sense of anonymity and gives an opportunity to participate in the study at a time convenient for the respondent, which was very useful during the COVID-19 pandemic.

The questionnaire was designed based on previous research related to eating habits in catering establishments [38,75,76]. The questionnaire was checked by means of a pilot study with 25 people. All problems were identified, and the questionnaire was completed and amended. It was estimated by the pilot test that completing the form would take each participant around 10–15 min.

The study protocol was approved by the Ethics Committee of the Institute of Human Nutrition Sciences of the Warsaw University of Life Sciences (no. 12/2021).

### 3.2. Questionnaire

The questionnaire structure for this study is presented in Table A1 (Appendix A). It consists of two parts, with the first part containing 24 questions relating to the impact of the pandemic on the use of food services by Polish consumers. The questions covered habits associated with the use of catering services before (four questions) and during the pandemic (twenty questions), and anxiety about using these services during this difficult time. The questions also concerned the eating habits of respondents, such as preparing meals at home, choosing the type of catering establishments and type of meals in gastronomy, the impact of the pandemic on their diet (limit or increases different food products). Questions were also related to consumers’ concerns about food service use during the pandemic. Do they limit use of food services? What is their opinion on protective practices utilization in catering establishments during a pandemic? The second part of the questionnaire included five questions related to respondent’s sociodemographic details (gender, age, education, dwelling place, financial situation).

### 3.3. Data Collection

The computer-assisted web-based interviewing (CAWI) method was used to collect all data [77,78,79]. The survey was conducted on a group of 1021 adult respondents in Poland. Inclusion criteria for respondents of the study were as follows:

1. Each respondent between 18 to 65 years old who agreed to participate in the survey was invited to complete the questionnaire.
2. Anyone who used catering establishments.

The respondents were free to participate in the research.

### 3.4. Data Analysis

The statistical analysis of the results was performed using Statistica software (version 13.3 PL; StatSoft Inc., Krakow, Poland).

The Kruskal–Wallis (K–W) test was used in the study to assess the influence of factors describing the population on the examined features. In situations where there are only two samples in the analysis, the K–W test was replaced by the U-Mann–Whitney test [80,81]. Significance of differences between the values was determined at a significance level of *p* < 0.05.

Cluster analysis was used to classify food service consumers and their concerns about the COVID-19 pandemic. The purpose of the analysis is to create groups of respondents with a homogeneous approach to the use of catering services during the COVID-19 epidemic and homogeneous concerns regarding the use of these services. The vast majority of variables are on the ordinal scale, with a few variables on the nominal scale and two on the ratio scale. The measure of similarity used in cluster analysis is the distance in a multidimensional coordinate system. Due to the qualitative nature of the variables, the analysis used the percentage discrepancy as a measure of distance. It is the quotient of the number of dimensions with inconsistent values and the number of all dimensions. When studying distances between clusters of multiple elements, it is also necessary to establish a method for calculating the distances of clusters. The study decided to use the complete linkage clustering method, also known as the farthest neighborhood method. The distance between clusters is the distance of the farthest elements of both clusters [82,83,84,85,86,87].

At the beginning of the analysis, the agglomeration method was used to detect highly correlated variables (questions), i.e., variables that carry the same information, and to reduce their number. Then, using a reduced number of variables, the respondents were divided into homogeneous groups. In the first step, the optimal number of clusters was determined by the method of agglomeration. In the second one, the k-means method was used to finally divide all consumers into homogeneous clusters. Due to the difficult interpretation of the obtained results, it was decided to create the smallest possible number of clusters ensuring a clear segmentation of the community. For each of the clusters, the medians were calculated and used to identify the characteristics of the groups.

## 4. Results

### 4.1. Characteristics of Respondents

The characteristics of the respondents are presented in Table 1. The study involved mainly women, with secondary or higher education, living in different types of dwelling places. The respondents were in the range of 18–65 years old, had access to a computer and the Internet, and had computer literacy skills.

### 4.2. Diet and Eating Habits Connected to Catering Services before and during a Pandemic

Most respondents (95%) used food services regularly prior to the pandemic, usually once a month (median 4), once every two-three months (median 3), or rarely (median 2). These were various types of catering establishments (Table 2). They most frequently used the services of pizzerias, restaurants, fast food establishments, cafes and bars (Table 2).

For statistically significant results of the K-W test, a post-hoc analysis was performed using the Dunn test. Its results indicate groups of people using gastronomy with different frequency.

Various types of catering establishments were used significantly more often by men and people aged 26–40. People aged 41–55 used canteens significantly more often. The use of catering establishments also depended on the education of the respondents. People with primary and vocational education chose fast food establishments significantly more often. People with secondary education usually chose pizzerias, kebab establishments, cafes, and bars. On the other hand, people with higher education chose canteens and restaurants significantly more often. The choice of catering establishments was also influenced by the financial status and dwelling place of respondents. People with very good income and living in the city with over 250,000 inhabitants used gastronomy services more often (Table 2).

A significant percentage of respondents (96.6%) prepared their own meals at home. The frequency of preparing meals by the respondents before the pandemic and during the pandemic did not change and was on average several times a week. Nearly 50% of respondents did not change their diet during the pandemic. About 20% of respondents started to take care of their diet and limit their consumption of sweets. This was indicated significantly more often by younger people (*p* = 0.0014) and people with secondary education (*p* = 0.01). About 11.5–13.5% of respondents were limiting consumption of meat, started to pay attention to the energy value of meals, with 6.4% limiting consumption of fats. Meat in the diet was significantly more often restricted by people living in cities of more than 250,000 inhabitants (*p* = 0.0305), with a very good financial situation (*p* = 0.0440), while people aged 18–25 (*p* = 0.0000), with secondary education (*p* = 0.0001) paid attention to the energy value of the diet significantly more often. About 20% of the respondents drank more alcohol, ate more sweets, and didn’t pay attention to the energy value of meals, and 10.9% of people consumed more fats and carbs. Alcohol consumption increased significantly more often among men (*p* = 0.0059), people with secondary education (*p* = 0.0034), those living in large cities (*p* = 0.0003), with a very good financial situation (*p* = 0.0131). On the other hand, people with a bad financial situation significantly more often did not pay attention to the caloric content of meals (*p* = 0.0023). A small percentage of respondents ate more vegan dishes (0.4%).

### 4.3. Use of Catering Services during a Pandemic by Respondents

Before the pandemic, nearly 90% of respondents regularly used gastronomy services both on-site, take-away (or drive-thru) (median 5), as well as ordering for home or work delivery (median 4) (Table 3). During the pandemic, 61.3% of respondents limited leaving home and kept it to a minimum. Quite a significant percentage of respondents (87.9%) used food services during the pandemic. However, a limitation in the use of catering services on the premises, and take-away (median 4) was observed in favor of home and work deliveries, medians 5 (Table 3).

The results of Dunn’s test of multiple comparisons that followed a K–W test revealed more detailed information of the effect of socio-economic variables on food services use.

The respondents were asked if they plan return to food consumption on site in a catering establishment after the pandemic, and indicated that they already do it while maintaining hygiene and distance (31.6%) or used it when there was a possibility (30.8%). That was significantly more often reported by people in aged 26–40, with higher education, living in cities with over 250,000 residents, and with a very good financial situation. Only 6.4% of the respondents indicated that they would return to gastronomy only when the pandemic is over. Approximately 31.2% had no opinion on this subject, which was probably caused by the dynamically changing situation in terms of the number of infections.

The most common reasons for limiting the use of gastronomy services were working or learning online, lower frequency of business meetings (40.5% of indications), limiting tourist activity such as traveling, sightseeing, or use of hotels (37.5%), limiting shopping in malls (37.2%), restricting attendance at cultural events such as cinema, theater, and concerts (32%), fear of COVID-19 (30.4%), financial considerations (26.7%), and the limitation of people’s movements at petrol stations, airports, railway stations (22.8%), discomfort related to the restrictions in the catering sector (21.8%), restrictions at special events (17.4%), and other (0.9%).

### 4.4. Factors of the Choice of Gastronomic Services by Respondents during a Pandemic

The most important factors in the choice of food services by respondents during the pandemic were: the quality of meals, price, delivery options and order fulfillment execution time (median 6, Table 4). A significant percentage of respondents (59.9%) reported that the quality of food services during the pandemic was provided at the highest level. About one third (33.6%) of respondents rather agreed with this statement, 20.2% agreed and 6.1% strongly agreed. The opposite opinion was held by 10.5% of the respondents, and 23.4% did not have an opinion on this topic.

During social isolation, the respondents most often ordered pizza (77.3% of responses), fast-food meals (52.5%), Asian cuisine meals (42.4%), sushi (37.6%), American cuisine meals (35.5%), promotional meals (31.9%), traditional lunch meals (28%), and pasta (25.6%). Meals such as salads and fit dishes, street food, Indian cuisine, special diet meals (vegan, gluten meals, light meals), and desserts were ordered rarely (12.6–17.7% of indications). A few people mentioned kebab, hummus, dumplings, Georgian or Turkish dishes, as well as ‘box diet’.

Special promotions, such as special discount coupons (20.5%), discounts (22.4%), and additional bonuses, such as two for the price of one (33.3%), encouraged respondents to use food services. However, nearly 50% of the respondents did not report using any promotions in food services during the pandemic.

The results of the K–W test (Table 4) indicate that gender, dwelling place and financial status rarely significantly affect the factors of catering establishments choice. The possibility of ordering special dishes, assurance hygienic practices and sense of safety are not differentiated by the analyzed factors.

Based on Dunn’s post-hoc tests, we can conclude that mainly the behaviors of respondents aged 25–40 and 55+ differed from the others. They chose restaurants less often because of the quality of the served dishes and more often than others because of the price, popularity of the restaurant and the possibility of delivery. The oldest people, on the other hand, more often followed the brand and less often the nutritional value of the dishes. Consumer education also often differentiates the factors of choosing eateries. People with primary and vocational education are less likely to pay attention to the price, sense of safety, assurance hygienic practices and the possibility of delivering the ordered dishes. Respondents with higher education more often pay attention to the quality of dishes, the brand and opinions about a restaurant. Consumers with secondary education more often than others pay attention to the time of order fulfillment. Generally, the higher the education, the more factors are important in choosing a gastronomic establishment. Gender differentiates the choices of dining establishments in a few cases. Women attach more importance to the nutritional value of dishes and the possibility of choosing vegan and vegetarian dishes, while men are more concerned with the quality of the dishes.

In order to divide consumers into homogeneous groups using catering services during the epidemic, the agglomeration method of cluster analysis was used. Initially, 63 variables were included in the analysis. As a result of the agglomeration, groups of strongly related variables were created and all of them were removed, leaving one variable. As a result of this operation, 16 unique variables remained in the analysis. Using collected variables, an agglomeration of cases (respondents) was carried out. The aim was to create fewer homogeneous groups of respondents. It was decided to create 5 clusters:

Cluster 1: Homemade food enthusiasts;

Cluster 2: Non-regular gastronomy customers;

Cluster 3: Occasional customers of gastronomy;

Cluster 4: Moderate enthusiasts of gastronomy;

Cluster 5: Gastronomy enthusiasts.

**Figure 1:**
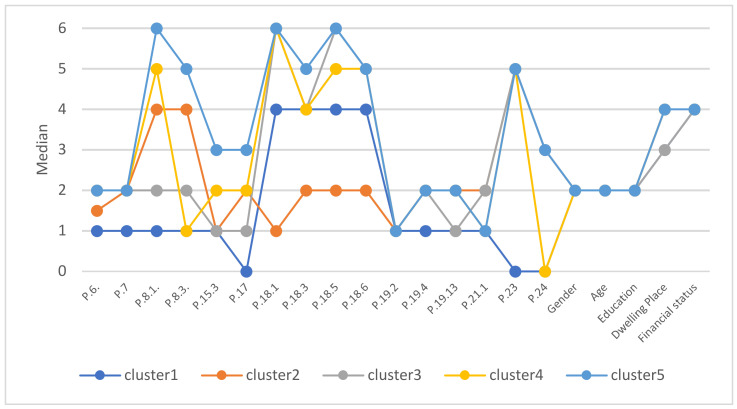
Medians of selected characteristics for observation in various clusters due to the behavior of consumers using catering services.

In the extended analysis, all observations were divided into 5 groups using the k-means cluster analysis method. Medians calculated for each variable (after reduction) and for each cluster were used to describe consumer behavior in individual clusters (Figure 1).

The first group of consumers (cluster 1—“Homemade food enthusiasts”) are people who did not use catering services during the pandemic (*n* = 101, 9.9% of respondents). These people also did not use often these services before the pandemic and do not plan to use them after the pandemic ends. The average representative of this group is a young woman (25–40 years old) with secondary education, living in a medium-sized city (50–250 thousand inhabitants), with a good financial situation. The lack of differentiation of the average consumer in clusters may indicate that the factors describing the respondents did not have a significant impact on their behavior as consumers of catering services.

Cluster 2 (“Non-regular gastronomy customers”) are people who use catering services (*n* = 94, 9.2% of respondents), but as a result of the pandemic, use them less often. Occasionally, they order food from eateries, sometimes through specialized portals. They don’t pay much attention to what they buy. They are generally satisfied with the services provided and plan to use them with appropriate precautions. These are the customers who use these services when they are unable to prepare their own meals.

Cluster 3 (“Occasional customers of gastronomy”) are consumers who use catering services very sporadically, rather conservatively, and not using specialized portals (*n* = 220, 21.5% of respondents). They attach great importance to all aspects of the catering service but are generally very satisfied with the services provided. They plan to return to gastronomy after the danger of the pandemic has ceased. These are occasional customers celebrating with purchased meals.

Cluster 4 (“Moderate enthusiasts of gastronomy”) consists of people who have reduced their use of catering services as a result of the pandemic, who order food moderately often but do not plan to eat at the premises even after the pandemic has stopped (*n* = 223, 21.8% of respondents). They attach great importance to all the features of the catering service, especially its quality and price. They order different dishes from time to time, depending on their mood or the food’s availability. They order food from catering establishments when they want a little variation in their daily diet.

Cluster 5 (“Gastronomy enthusiasts”) is a group of people who, despite the fact that they limited the use of catering services as a result of a pandemic, often order take-out food and often, if possible, use the services at the establishments (*n* = 383, 37.5% of respondents). They quite often use the help of specialized gastronomic portals and pay for orders using electronic payments. They value the high quality and safety of catering services, but do not neglect its other aspects as well. They do not have specific tastes regarding their selected dishes, but rather avoid traditional cuisine. They are generally satisfied with these services and use them consistently, declaring safety as an important behavior.

### 4.5. Respondents’ Sense of Security When Using Catering Services during a Pandemic

During the pandemic, both in the period of no possibility to consume in catering establishments and after the opening them, the respondents indicated that their use of food services sometimes raised anxiety (median 2—yes). After the opening of catering establishments, this was indicated significantly more often by people in large cities over 250,000 inhabitants (*p* = 0.0011) and people with higher education (*p* = 0.0281). The smallest number of respondents (58.3%) reported that they felt safe on the premises. Choosing take-away option (69.3%), ordering home or work delivery (72.2%), collecting ordered meals from catering establishments themselves was according to respondents safer. The most important forms of protection customers who consumed foods on the premises during the pandemic were considered: hand disinfection (70.3% of indications), disinfection of tables (70.4%), wearing of masks and the visors by staff (68.2%) and the possibility of cashless payments (Table 5). The same forms were indicated during the purchase of take away or drive-thru, as well as home and work deliveries.

Many respondents stated that in numerous establishments no safeguards were introduced. The proper distance was not kept, there were too many people on the premises, masks were not used by personnel and customers or were worn incorrectly, no gloves were used, no hand disinfection was used, and finally the payment terminals were not disinfected. Most comments were made about the disinfection of the tables. According to the respondents, in many places the tables were not disinfected at all or inadequately, or even only a misleading or false note about disinfection was on the table.

### 4.6. Consumer Segmentation Due to Concerns about Food Service Use during a Pandemic

For the cluster analysis the concerns of consumers using gastronomy during the epidemic, fifteen variables describing consumer behavior and five variables describing consumers were used. As a result of the agglomeration of variables, strongly related variables were removed, leaving one representative for each group of related variables. The variables Q9.1, Q.10.1, Q.25.4 and Q.25.5 and the variables describing the respondents remained in the analysis. Based on the cases agglomeration diagram, the optimal number of clusters was determined to be eight. The physical division of the sample into clusters was performed using the k-means method. The values of medians calculated for each of the variables in the cluster were used to describe the cluster.

**Figure 2:**
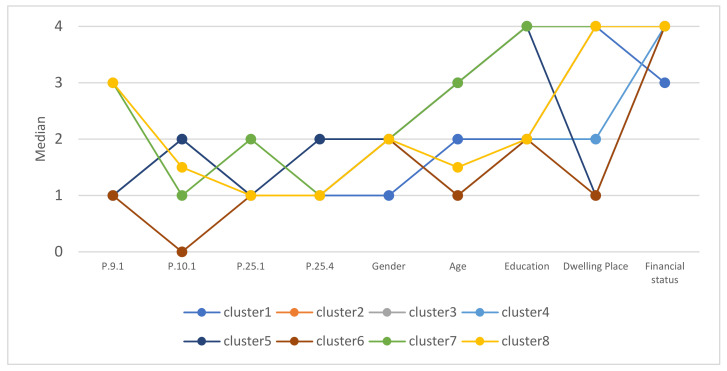
Medians of selected characteristics for observations in different clusters due to both related to the epidemic.

On the basis of the median values (Figure 2), it can be concluded that the group consisting of clusters from 1 to 4 are people who do not have any anxiety using gastronomy during an epidemic (Table 6). These are people who were not afraid of using catering services, felt safe while using them both inside and outside the premises, did not limit the activities during which you can use catering services, and were not afraid of infection by COVID-19. Cluster 5 consisted of people who do not have concerns about the use of gastronomy, but had to limit the use of food services due to the introduced restrictions. Clusters 6–8 grouped people who were afraid of using food services during the pandemic and were restricting use of services due to concerns about getting sick. It was their intentional and conscious action.

Based on segmentation, it can be concluded that the majority of respondents do not see any threats to the use of catering services, do not care about potential problems, and do not intend to change their approach to this type of service due to concerns about the possibility of getting sick with COVID-19.

### 4.7. The Use of Food Delivery from Gastronomy to Home by the Respondents

A significant percentage of respondents used home delivery food services (87.5%). The respondents most often used direct food delivery from catering establishments (58.1%), which they used rarely more than once every two or three months. The other internet portals or applications respondents used them sporadically during the year. Respondents reported that they used apps such as Uber Eats (34.4%), Glovo (19.1%), and Polish equivalent of apps Just Eat Takeaway.com (49.9%), Bolt Food (9.4%), Wolt (7.1%), and local on-line delivery food platforms (11.7%). Using food delivery to home was dependent on gender, age, education, dwelling place, and financial status (*p* < 0.005). Using local on-line delivery food platforms depended only on gender (*p* = 0.029). This delivery form was usually used by young people up to 25 years old. Payments were usually made online (43.9%) or by credit card (35.8%). However, 11.7% of people paid for the delivery in cash.

During delivery, the supplier handed over the order from a distance while maintaining the sanitary regime (59.2% of indications), or delivered and left it at the door (22.3%). A small percentage (5.9%) of the study participants reported hygiene non compliances during food delivery.

## 5. Discussion

The obtained results indicate a change in eating habits and consumer behavior related to the use of catering services by Poles. This is interesting because, despite the prevailing pandemic, there is no evidence that COVID-19 is transmitted through food consumption [88,89,90,91,92]. However, infection can occur by inhaling the virus within 1 m of a COVID-19 infected person, or by touching a contaminated surface and then touching the eyes, nose, or mouth before washing hands [91]. For this reason, eating in restaurants during an outbreak can be viewed as high risk due to the possibility of infection in a closed dining room and contact with service providers and other customers.

According to this research, respondents limited the use of on-premises catering services and preferred the option of ordering take-away food, food delivery for home and work, or a drive-thru (after the opening of catering establishments during pandemic). Yang et al. [53] found that a 1% increase in daily new COVID-19 cases led to a 0.0556% decrease in restaurant demand while stay-at-home orders were associated with a 3.25% demand decline.

In March and April 2020, restaurants in the U.S. recorded a significant increase in orders for food delivery on the Uber Eats platform [93], and these consumers (who were highly afraid of the COVID-19) chose a private dining restaurant and a private dining table [51]. Mobility restrictions, the lockdown, and the closure of catering establishments have led to a sharp decline in consumer demand for lunch restaurants. The number of consumers eating lunches in gastronomy fell in March 2020 to the lowest level in history in many countries due to strict sanitary rules [37]. Another reason could be that consumers are more likely to limit their spending on meals outside the home, because cooking at home, especially for larger households, is cheaper [53,94]. Moreover, studies have shown that consumers had more time to prepare meals during the pandemic [60,62].

As shown, among the 8 clusters selected in the analyses in terms of respondents’ concerns about the pandemic, only a small group of people (8.1%) feared for their health while using food services during the pandemic. Nevertheless, they limited use of food services due to work or online learning and limited some activities, including tourism and entertainment.

The respondents mentioned the quality of the dishes, the price, the possibility of delivery, and delivery time as the most important factors in choosing catering services during the pandemic. The quality of services provided during the COVID-19 epidemic may result in increased loyalty to catering establishments after its end [22]. It is interesting that maintaining the sanitary and hygienic regime were mentioned subsequently. This may be due to the fact that the pandemic has forced people to eat their food off-site from establishments and caused many critical changes to food services. In response to the COVID-19 crisis, gastronomy has adapted various operating strategies, including changes in service delivery methods [95,96], adoption of new technology [97], and strict compliance with hygiene and safety standards [98].

Catering establishments have implemented alternative models of providing services outside the premises, such as drive-thru and direct food delivery or ordering using an application and delivery through external companies such as Uber Eats, Bolt, and others. Non-cash payment and sanitary procedures have been implemented to make consumers feel safe, so that they did not have to give up their eating habits. However, these service models often provide an advantage to fast food restaurants that already had digital infrastructure and drive-thrue. Not all full-service restaurants were able to quickly adapt to change [99]. The COVID-19 pandemic differs from other food organization crises because of its severity, devastating effects, evolving nature, and the very limited way organizations were able to control the situation. The catering sector faced new challenges, starting with disruptions in the supply chain, changing market demand, maintaining a high level of food safety, and maintaining consumer confidence [92,100,101,102,103,104,105].

Respondents were good observers of maintaining hygiene and ensuring consumer safety during a pandemic, both while staying at the premises and ordering food for home or work. Consumers’ sense of security when using catering services is associated with the perception of the risk of contracting the COVID-19 virus. It should be remembered that the lack of safeguards may deter consumers from eating during a pandemic where there is a high risk of infection [52]. The respondents considered the following the most important forms of protection during the pandemic: hand disinfection (70.3%), table disinfection (70.4%), wearing of masks and visors by staff (68.2%), the possibility of making cashless payments (64.6%), and maintaining social distance (59.2%). According to studies [7,89,91,106,107], washing and disinfection of hands by staff and customers, wearing disposable gloves by staff, as well disinfection of all contact surfaces (dishes, trays, tables, handles, doors, chairs, dispensers) are the most important and critical activities undertaken in order to limit the spread of the virus and avoid disease [7,89,91,106,107]. Catering establishments should also install plexiglass partitions in order to increase the distance and separate employees from guests or between consumers [108]. It should be emphasized that, unfortunately, in many places these rules were not applied, which was pointed out by the respondents.

During the pandemic, respondents most often used pizzerias, fast food establishments, restaurants and cafes, followed by establishments offering Asian food and kebab bars. These are the establishments that Polish consumers also used most often before the pandemic. Fast-food restaurants offer more options for take-away service that enable consumers to take advantage of the service without staying on the premises. Yang et al. [36] found a lower negative impact of COVID-19 when using nutrition in fast food restaurants compared to full-service establishments. A full cycle of waiter service in restaurants can increase consumers’ perception of contagion risk. Consumers may therefore have a different perception of the contagion risk related to eating meals in fast-food restaurants compared to full-service restaurants [36]. COVID-19 disease can be spread through physical contact, which is limited in fast food restaurants, which may contribute to the perception of lower risk in these establishments. The ongoing COVID-19 pandemic and related global restrictions have caused an economic crisis, and consumer spending has largely decreased accordingly [109]. Hence, consumers are more likely to avoid gourmet meals and look for cheaper options of meals, such as fast food. People with low incomes, and with low education, mainly use fast-food establishments. The restaurants are chosen mainly by people with average and high income which is usually connected with high education [72,110,111].

Only half of the respondents reported changes in their diet due to the pandemic. They included greater attention to their diet, limited consumption of sweets, meat, and fats, and reduced caloric value of meals. However, 20% of respondents reported drinking more alcohol, eating more sweets, not paying attention to the energy value of meals, and consuming more fats and carbs. The nutritional changes, both positive and negative, also indicated by other authors [66,112]. Similar results were stated in Italy. There was an improvement in the consumption of components of the nutritional pattern in the Mediterranean population. Natural products such as garlic, sage, and olive oil have been proposed that are inherent in this diet as additional measures to prevent and treat COVID-19 [61,66].

Similar relationships are also indicated by other authors, including an increase in consumption of animal products and snacks [53,113], increasing salty snacks [32], increasing fat consumption [31], increasing ‘junk food’ consumption [61], snacking between meals [59], increased use of alcohol [65], and increasing sweets [34,65], especially during the lockdown. Authors [35,69,114] indicated that the stress associated with pandemic influenced on increases in emotional food of consumption, tasty but with high energy value. It should be emphasized that poor food choices in the long term could result in increased risk factors for cardiovascular diseases, diabetes, and cancer [115]. Many authors also noted positive changes, such as an increase in the consumption of vegetables and fruits, legumes, and fish [32,33,63,67]. At the beginning of the COVID-19 pandemic, some authors [31,68,116,117,118,119] have found an increase in the consumption of long-life foods, and a decrease in the consumption of fresh-from-food. Authors [68,119] explained that the limitation of vegetable and fruits consumption was connected with its low quality, poor availability, high price, and decrease frequency of shopping by consumers.

Among the positive effects of the pandemic in Polish gastronomy, there was an increase in interest in catering for all-day meals for private individuals (the so-called box diets). These types of meals are often of a dietary nature or are profiled for specific recipients (physically active people, athletes, vegans, etc.) [18].

### Limitations

This study has some limitations in terms of both its methodology and its applica-bility. The sample selected for the study consisted mainly of people between 18 and 55 years old; therefore, caution should be exercised in attempting to extrapolate the results to an entire population. In addition, the study was conducted mainly in large Polish cities. Consumers’ behavior may be different in other places. Another limitation is that the respondents were from only one country. Despite the limitations, the results obtained are of practical importance, and demonstrate the nutritional behavior of consumers during a pandemic, especially in catering establishments.

## 6. Conclusions

Almost half of the respondent group did not change their diet during the pandemic. About 20% of people began to pay attention to their diet, especially to the energy value of meals, and limited the consumption of sweets, meat, and fats. About 20% of respondents drank more alcohol, ate more sweets, consumed more fats and carbs, and didn’t pay attention to the energy value of their diet. They also did not change their habits of preparing home meals and prepared them several times a week. It was found that COVID-19 had an impact on consumers’ use of food services, both on-site and take-away. Due to the online mode of work and teaching, as well as the limitations on tourist and entertainment activities, the use of restaurants was limited in favor of home and work deliveries. The type of dishes ordered by respondents did not change and was similar to before the pandemic. Five consumer clusters were identified, differing in terms of the frequency of using various forms of catering services, including specialist portals and food ordering applications.

A majority of respondents (about 70%) considered the following as the most important forms of protection during the pandemic when using food services, hand disinfection, table disinfection, wearing of masks and visors by staff, and the possibility of cashless payments. However, respondents indicated that their use of gastronomic services sometimes raised concerns due to non-compliance with hygiene rules in establishments. Due to concerns about using catering services, eight consumer clusters were identified. Only two clusters of respondents who were concerned about the safety of food service and consciously limited use of these services were selected. The pandemic has affected eating behavior of respondents in catering establishments but have not changed the consumers’ habits. It is worth paying attention to further research on the subject. Researchers conducting their studies in different countries will be able to better understand the consequences are of the pandemic in gastronomic sector but their inhabitants will have different food preferences and thus their behavioral changes may differ from herein.

## Figures and Tables

**Table 1:** Characteristics of the surveyed sample of respondents.

Population Features	Group	Respondents	
		Number (*n*)	Percentage (%)
Total	--	1021	100.0
Gender	women men	658 363	64.4 35.6
Age	18–25 years old 26–40 years old 41–55 years old >56 years old	364 398 228 31	35.7 39.0 22.3 3.0
Education	vocational or primary school secondary school higher education (university)	152 485 384	14.9 47.5 37.6
Dwelling place	city over 250,000 inhabitants city between 50,000–250,000 inhabitants) city between 10,000–50,000 inhabitants city below 10,000 inhabitants and village	543 131 164 183	53.2 12.8 16.1 17.9
Financial situation in own opinion	very good good not good not bad bad	163 541 261 56	16.0 53.0 25.5 5.5

**Table 2:** Using catering establishments, frequency of use before pandemic time.

Type	Response		Frequency of Use **	*p*-Value *				
	Number	Percentage	Median	Gender	Age	Education	Dwelling Place	Financial Status
1. Canteens	759	74.3	2	0.0000	NS	0.0109	0.0000	0.0078
2. Fast food	900	88.1	4	0.0007	0.0000	0.0000	NS	NS
3. Restaurants	913	89.4	4	NS	0.0000	0.0000	0.0000	0.0000
4. Pizzerias	943	92.4	4	0.0102	0.0000	0.0002	NS	0.0005
5. Kebab house	853	83.5	3	0.0000	0.0000	0.0005	NS	NS
6. Asian food restaurants	823	80.6	3	0.0000	0.0000	0.0233	0.0000	0.0007
7. Café and bars	868	85.0	4	NS	0.0006	0.0000	0.0002	0.0001
8. Roadside catering	804	78.7	2	0.0067	0.0052	NS	NS	0.0133
9. Street food outlets	717	70.2	2	0.0119	0.0000	NS	NS	0.0044

* significance values in the ANOVA K–W tests, NS—not significant, *p* < 0.05; ** (8): every day; (7): three or four times a week; (6): once a week; (5): two-three times a month; (4): once a month; (3): once 2–3 months; (2) rarely than once 2–3 month; (1) did not use.

**Table 3:** Using catering establishments and frequency of use by respondents before and during pandemic.

Place	Response		Frequency of Use **	*p*-Value *				
	Number	Percentage	Median	Gender	Age	Education	Dwelling Place	Financial Status
Before the pandemic								
On-site/dine in	938	91.9	5	NS	0.0001	0.0002	NS	0.0158
Take away/drive thru	887	86.9	5	0.0374	0.0002	0.0344	NS	0.0391
Home/work delivery	843	82.6	4	0.0000	0.0000	0.0002	0.0000	0.0013
During the pandemic								
On-site/dine in	728	71.3	4	0.0005	0.0002	0.0000	0.0000	0.0000
Take away/drive thru	771	75.5	4	0.0001	0.0000	0.0085	0.0014	0.0005
Home/work delivery	810	79.3	5	0.0664	0.0036	0.0008	0.0000	0.0000

* time significance values in the ANOVA K–W tests; NS—no significant, *p* < 0.05; ** (8): every day; (7): three or four times a week; (6): once a week; (5): two-three times a month; (4): once a month; (3): once a 2–3 months; (2) rarely than once a 2–3 month; (1) Did not use.

**Table 4:** Factors of catering establishments choice by respondents during a pandemic.

Factors of Choice	Response **	*p*-Value *				
	Median	Gender	Age	Education	Dwelling Place	Financial Status
Quality of dishes	6	0.0788	0.0000	0.0000	0.0002	0.0000
Price	6	NS	0.0001	0.0001	0.0016	NS
Brand	4	NS	0.0429	0.0081	NS	0.0001
Opinion and popularity of the premises	5	NS	0.0000	0.0000	0.0313	NS
Sense of safety	5	NS	NS	0.0046	NS	NS
Nutritional value	4	0.0077	0.0129	NS	NS	0.0077
Possibility to order meals: vegan, low fat, gluten free	4	0.0001	NS	NS	NS	NS
Assurance hygienic practices	5	NS	NS	0.0031	NS	NS
Order fulfillment time	6	NS	0.0133	0.0010	NS	NS
Possibility of delivery	6	NS	0.0005	0.0002	NS	NS

* significance values in the ANOVA K–W tests, NS-no significant; ** Scale: (1): strongly disagree; (2): disagree; (3): somewhat disagree; (4): neither agree nor disagree; (5) somewhat agree; (6) agree: (7) strongly agree.

**Table 5:** Protection used in catering establishments against the spread of the virus *.

Protection Forms	Dine-in		Take Away/Drive-thru		Home/Work Delivery		Security Validity *	
	n	%	n	%	n	%	Average	Median
Distance of 2 m from other people	604	59.2	403	39.5	415	40.6	3.7	4
Plexiglass partitions	429	42.0	323	31.6	59	5.8	3.4	3
Hand disinfection	718	70.3	380	37.2	231	22.6	4.3	5
Staff wearing disposable gloves	577	56.5	544	53.3	442	43.3	4.0	4
Staff wearing protective masks or visors	696	68.2	596	58.4	617	60.4	4.3	5
Disinfection of tables	719	70.4	117	11.5	76	7.4	4.3	5
Cashless payment availability	660	64.6	611	59.8	640	62.7	4.1	4
Disinfection of payment terminals	359	35.2	257	25.3	174	17.0	3.7	4
Other non-compliances	71	7.0	23	2.3	33	3.2	-	-

* (5): very important; (4): important; (3): neutral; (2): low importance; (1): not at all important.

**Table 6:** Cluster analysis results for consumer anxiety about the use of food services during a pandemic.

Cluster	Number of Respondents (%)	Characteristic of an Average Representative	Anxiety
Cluster 1	220 (21.5%)	young men (26–40 years old), with secondary school education, living in large cities (>250,000 inhabitants), with a good financial situation	They haven’t
Cluster 2	167 (16.4%)	young women (18–25 years old), with secondary school education, living in large cities (>250,000 inhabitants), with a good financial situation	They haven’t
Cluster 3	110 (10.8%)	women 41–55 years old, with higher education, living in large cities (>250,000 inhabitants), with a good financial situation	They haven’t
Cluster 4	187 (18.3%)	young women (18–25 years old), with secondary school education, living in village and city below 10,000 inhabitants, with a good financial situation	They haven’t
Cluster 5	85 (8.3%)	women 41–55 years old, with higher education, living in village and city below 10,000 inhabitants	They haven’t, and they did not change the behavior in gastronomy, except for those related to the participation in sports, cultural and other events (administrative restrictions).
Cluster 6	87 (8.5%)	young women (18–25 years old), with secondary school education, living in village and city below 10,000 inhabitants	No opinion about the safety risks when using the food services. They did not change their behavior because they didn’t orientate in pandemic topic.
Cluster 7	83 (8.1%)	women 41–55 years old, with higher education, living in big cities (>250,000 inhabitants and city between 50,000–250,000 inhabitants)	They are concerned about the safety of food services (aware of the dangers)
Cluster 8	82 (8.0%)	young women (18–25 and 26–40 years old) with secondary school education, living in large cities (>250,000 inhabitants)	They are concerned about the safety of food services and do not use them in an epidemic, but also rarely used them before a pandemic

## Data Availability

The data presented in this article is available on reasonable request, from the corresponding author.

## Appendix A

**Table A1:** Questionnaire structure.

Question	Variants of Answers
Q1. Did you use catering services before the pandemic?	Choose the right answer: (1): yes, (2): no
Q2. How often (before the pandemic) did you use a particular category of catering services? Q2.1. on-site/dine in, Q2.2. Take-out/drive-thru, Q2.3. Home/work delivery	(8): every day, (7): three or four times a week, (6): once a week, (5): two-three times a month, (4): once a month, (4): once a 2–3 months, (2): rarely than once a 2 a 2–3 month, (1): I did not use
Q3. Which catering establishments did you use (before the pandemic) and how often? Q3.1. canteens, buffets, Q3.2. fast-food, Q.3.3. full-service restaurants, Q3.4. pizzerias, Q3.5. kebab house, Q3.6. Asian restaurants, Q3.7. cafes and bars, Q.3.8. motorway service station, Q3.9. street-food vendors	(8): every day, (7): three or four times a week, (6): once a week, (5): two-three times a month, (4): once a month, (3): once a 2–3 months, (2) rarely than once a 2–3 month, (1) I did not use
Q4. Do you prepare meals at home?	Choose the right answer: yes (1), no (0)
Q5. How often do you cook at home? Q5.1. before pandemic, Q5.2 during a pandemic	(8): every day, (7) three or four times a week,(6) once a week, (5) two-three times a month, (4) once a month, (3) once a 2–3 months, (2) rarely, (1) I did not use
Q6. Do you refrain from leaving home to a minimum during the pandemic?	Choose the right answer: (1): yes, (2): no
Q7. Do you use catering services during a pandemic?	Choose the right answer: (1): yes, (2): no
Q8. If yes, please specify which category and how often do you use them? 8.1. home/work delivery, 8.2. Take away/drive thru, 8.3. on-site (after opening the premises)/dine in	(8): every day, (7) three or four times a week, (6) once a week, (5) two-three times a month, (4) once a month, (3) once a 2–3 months, (2) rarely, (1) I did not use
Q9. Were you afraid to use catering services? Q9.1. period when consumption on-premises was not possible, Q9.2. period after the opening of the dining area on-premise.	(4): always, (3): often, (2): sometimes, (1): never
Q10. Did you feel safe using various catering services? Q10.1. Home/work delivery, Q10.2. take away/drive thru, Q10.3. On-site (after opening the premises)/dine in	Choose the right answer: (1): yes, (2): no, (0): I have no opinion
Q11. What type of safety practices have been used in catering services? Q11.1 Distance of two meters from other people, Q11.2. Plexiglass partitions, Q11.3. hand disinfection, Q11.4. staff wearing disposable gloves, Q11.5. staff wearing protective masks or visors, Q11.6. disinfection of tables, Q11.7. cashless payment availability, Q11.8. disinfection of payment terminals, Q11.9. other non-compliances	Choose the right answer: (1) Home/work delivery, (2) take out/drive-thru, (3) on-site (after opening the premises)/dine in
Q12. What other safety non compliances were found while using catering services?	Please specify
Q13. Did you pick up the order from the restaurant by yourself?	Choose the right answer: (1) yes, (2) no
Q14. What protective practices were important for you when preparing/serving/consuming a meal on-site in a catering establishment? Q14.1. Distance of 2 m from other people, Q14.2. Plexiglass partitions, Q14.3. hand disinfection, Q14.4. staff wearing disposable gloves, Q14.5. staff wearing protective masks or visors, Q14.6. disinfection of tables, Q14.7. cashless payment availability, Q14.8 disinfection of payment terminals, Q14.9. Other (Please specify).	Choose the right answer: (5): very important, (4): important, (3): neutral, (2): low importance, (1): not at all important
Q15. What online food delivery platforms/mobile apps did you use the during the pandemic?: 15.1: Uber Eats, 15.2: Glovo, 15.3: Just Eat Takeaway.com (in Poland Pyszne.pl), 15.4: Smacznie i szybko.pl, 15.5: Głodny.pl, 15.6: Pizza.pl, 15.7: Bolt Food, 15.8: Wolt, 15.9: Delivery from establishments, 15.10: Other (Please specify)	(6) every day, (5) three or four times a week, (4) once a week, (3) two-three times a month, (2) once a month, (1): not once
Q16. What delivery methods did you usually use?	(1): delivery and leave food at the door, (2): delivery food with hygienic rules, (3): other non compliances
Q17. How did you usually make the payment?	Using: (1) a payment card, (2) cash, (3) pre-paid
Q18. What did you pay attention to when ordering during the pandemic? Q18.1. quality of dishes, Q18.2. price, Q18.3. brand, Q18.4. opinion and popularity of the establishment, Q18.5. sense of security, Q18.6. nutritional value, Q18.7. possibility to order meals: vegan, low fat, gluten free, Q18.8. assurance hygienic practices, Q18.9. order fulfillment time, Q18.10. delivery availability	(1): strongly disagree, (2): disagree, (3): somewhat disagree, (4): neither agree nor disagree, (5) somewhat agree, (6) agree, (7) strongly agree
Q19. What dishes and how often did you usually order during the pandemic? Q19.1. Special offer menu items, Q19.2. Traditional lunch meals, Q19.3. American Cuisine, Q19.4. Pizza, Q19.5. Salads and fit meals, Q19.6. Pastas, Q19.7. Sushi, Q19.8. Asian cuisine, Q19.9. Street food, Q19.10. Special Diet Meals (e.g., gluten free), Q19.11. Indian cuisine, Q19.12. Desserts, Q19.13. Fast-food, Q19.14. Other (Please specify)	(1): never, (2): once or two a month, (3): three-four times a month, (4): once a week, (5): two or three times a week, (6): every day
Q20. Have you noticed any special offers when ordering meals during the pandemic? Multiple choice answers	(1): special discount coupons, (2): discounts (e.g., −50%), (3): freebies, bonuses (e.g., beverage for free, buy one, get one free), (4): I have not noticed, (5): other (please specify)
Q21. Have your diet changed during the pandemic? Multiple choice answers	(1): It did not change, (2): I started to take care of my diet, (3): I have eaten more vegetarian dishes, (4): I have limited sweets consumption, (5): I have limited meat consumption, (6): I have limited fat consumption, (7): I have paid attention to the caloric value of the meals, (8): I have not paid attention to the caloric value of the meals, (9): I have eaten more sweets, (10): I have drunk more alcohol, (11): I have consumed more fat/carbs
Q.22. Do you agree with the statement: ‘The quality of catering services during the pandemic was provided at the highest level’	(1): strongly disagree, (2): disagree, (3): somewhat disagree, (4): neither agree or disagree, (5) somewhat agree, (6) agree, (7) strongly agree
Q23. When do you intend to use the catering services on the restaurant premise?	(1): When the pandemic will end, (2): When the daily number of new COVID-19 cases in my region/city will be close to zero, (3): I do not know, (4): I did it as soon as it was possible, (5): I use it respecting the principle of hygiene and social distancing
Q24. If you have limited the frequency of using catering services, please indicate the reasons for your decision.	(1): Fear of COVID-19 infection, (2): Financial reasons (reduction of income, savings), (3): Work or remote learning (less frequency of business meetings, online classes), (4): Limited tourist activity (traveling, sightseeing, using hotels), (5): Limited activity in cultural events (cinema, theater, concerts), (6): Limited activity in shopping centers, (7): Restriction of movement (petrol stations, airports, railway stations), (8): Discomfort related to the restrictions in catering industry, (9): Limitation in the organization of social event catering (e.g., wedding), (10): Other (Please specify).
